# Effectiveness, safety, and acceptability of first‐trimester medical termination of pregnancy performed by non‐doctor providers: a systematic review

**DOI:** 10.1111/1471-0528.14712

**Published:** 2017-08-17

**Authors:** S Sjöström, M Dragoman, MS Fønhus, B Ganatra, K Gemzell‐Danielsson

**Affiliations:** ^1^ Department of Women's and Children's Health Karolinska Institutet Karolinska University Hospital Stockholm Sweden; ^2^ UNDP/UNFPA/UNICEF/WHO/World Bank Special Programme of Research Development and Research Training in Human Reproduction (HRP) Department of Reproductive Health World Health Organization Geneva Switzerland; ^3^ Norwegian Institute of Public Health Oslo Norway

**Keywords:** Incomplete abortion, medical termination of pregnancy, midlevel providers, non‐doctor providers, systematic review

## Abstract

**Background:**

Previous systematic reviews have concluded that medical termination of pregnancy (TOP) performed by non‐doctor providers may be as effective and safe as when provided by doctors. Medical treatment of incomplete miscarriage by non‐doctor providers and the treated women's acceptance of non‐doctor providers of TOP has not previously been reviewed.

**Objectives:**

To review the effectiveness, safety, and acceptability of first‐trimester medical TOP, including medical treatment for incomplete miscarriage, by trained non‐doctor providers.

**Search strategy and selection criteria:**

A search strategy using appropriate medical subject headings was developed. Electronic databases (PubMed, Popline, Cochrane, CINAHL, Embase, and ClinicalTrials.gov) were searched from inception through April 2016. Randomised controlled trials and comparative observational studies were included.

**Data collection and analysis:**

Meta‐analyses were performed for included randomised controlled trials regarding the outcomes of effectiveness and acceptability to women. Certainty of evidence was established using the GRADE approach assessing study limitations, consistency of effect, imprecision, indirectness and publication bias.

**Main results:**

Six papers were included. Medical TOP and medical treatment of incomplete miscarriage is probably equally effective when performed by non‐doctor providers as when performed by doctors (RR 1.00; 95% CI 0.99–1.01). Women's acceptance, reported as overall satisfaction with the allocated provider, is probably equally high between groups (RR 1.00; 95% CI 1.00–1.01).

**Conclusion:**

Medical TOP and medical treatment of incomplete miscarriage provided by trained non‐doctor providers is probably equally as effective and acceptable to women as when provided by doctors.

**Tweetable abstract:**

Medical termination of pregnancy performed by doctors and non‐doctors can be equally effective and acceptable

## Tweetable abstract

Medical termination of pregnancy performed by doctors and non‐doctors can be equally effective and acceptable.

## Introduction

Unsafe termination of pregnancy (TOP) is the cause of substantial maternal mortality and morbidity worldwide. Factors such as legal restrictions and stigma aggravate estimates, but around 25% of all pregnancies are assessed to be terminated, and at least 22 800 preventable deaths occur globally each year.^1^, ^2^

The scarcity of healthcare providers is one of many recognised barriers to safe TOP.^3^ Legal regulations limiting TOP provision to specialist doctors and a reluctance to provide TOP among potential healthcare providers because of stigma and fear of reprisals are among reasons that providers of TOP, and especially medical TOP, are limited in higher as well as lower resource settings, even where TOP is legal.^4^, ^5^, ^6^ Women's preference of providers influence their care‐seeking behaviour, and may therefore increase their risk of undergoing unsafe procedures.^7^ Acceptability, womens' ability to accept aspects of care, and satisfaction with services are particularily important in settings where women are at risk of unsafe TOP.^8^

Task shifting and sharing of medical services with trained non‐doctor providers has the potential to increase access and decrease unsafe TOP, and it's consequences. Previous reviews have found that non‐doctor provision of TOP is efficacious and safe, but those reviews included studies on surgical TOP and a limited number of studies on medical TOP.^9^, ^10^, ^11^ The World Health Organization (WHO) has recently developed guidelines recommending that medical TOP and treatment for incomplete miscarriage in the first trimester using recommended clinical interventions (vacuum aspiration and medical TOP using mifepristone and misoprostol, or misoprostol alone, as well as medical treatment for incomplete miscarriage using misoprostol) can be managed by trained auxiliary nurse midwives, nurses, midwives, and associate clinicians.^12^, ^13^ Treatment for incomplete miscarriage with misoprostol is an acceptable alternative to surgical evacuation.^14^ Medical methods are especially feasible in settings with limited healthcare facilities, but is often not offered because of legal restrictions and a lack of knowledge and training among existent providers.^15^, ^16^

There is growing evidence supporting that provision of medical TOP and treatment of incomplete miscarriage by trained non‐doctor providers is as effective and safe as provision by doctors, as well as being cost‐effective.^17^ The provision of either treatment involves similar skills in terms of eligibility assessment, counselling, administration of medication, and assessment of completion. This study aims to review the effectiveness, safety, and acceptability of non‐doctor provision of first‐trimester medical TOP, including medical treatment for incomplete miscarriage.

## Methods

### Inclusion criteria

The criteria for considering studies for review were defined in terms of PICOs (participants, interventions, comparisons, outcomes, and study designs) questions. Participants were pregnant women seeking medical TOP through 12 weeks of gestation or medical treatment for incomplete miscarriage (including both miscarriage and TOP). The intervention was medical TOP or medical treatment of incomplete abortion provided by non‐doctor providers [auxiliary nurse midwives (ANMs), nurses, midwives, non‐conventional therapies doctors or associate clinicians]. Comparisons were medical TOP or medical treatment of incomplete miscarriage provided by non‐specialist and specialist doctors. The outcome measures were:

- effectiveness, measured as complete TOP without need for additional surgical intervention following the procedure;
- safety, measured as serious adverse events (need for hospital admission, blood transfusion, or death);
- acceptability, measured as women's overall satisfaction with the provider or services provided, and whether women would recommend the same treatment or provider to a friend, or whether they would prefer the same type of provider in the case of a future termination of pregnancy.

Eligible study designs were randomised controlled trials (RCTs) and comparative observational studies, including cohort and case–control studies.

### Search strategy

A search strategy was developed using relevant medical subject headings (MeSH) and free‐text words (tw) for each of the study components and adapted to all included databases. We combined search terms for provider types and TOP generally, as well as specific tasks associated with the process (Appendix S1). The databases PubMed, EMBASE, CINAHL, POPLINE, Global Index Medicus, Cochrane database, and ClinicalTrials.gov were searched from inception through July 2014 for all articles published in peer‐reviewed journals, and the search was updated using the same search strategy in April 2016. There were no time or language restrictions.

### Data collection and analysis

Two authors (MD and SS) reviewed the titles and abstracts and, when necessary, the full article to identify studies that met the PICOs criteria. Reference lists from articles identified by the search, as well as other key reviews, were hand‐searched to identify additional papers. We also contacted investigators with continuing trials identified on ClinicalTrials.gov and other researchers in the field to seek information on unpublished or continuing studies that were unavailable through the electronic search.

We systematically and independently abstracted and summarised the evidence (MD and SS) using standard abstraction forms considering study characteristics, including design, participants, intervention, outcome, and assessment method. We (MD and SS) assessed the risk of bias in individual studies based on the criteria outlined in the Cochrane Handbook for Systematic Reviews of Interventions.^18^

Results for our outcomes were pooled in meta‐analyses by one researcher (MSF) using revman 5.3 (2014).^19^ The measures of effect were pooled risk ratios (RRs) of the outcomes. Data on the number of events and number of participants assigned to each treatment group were meta‐analysed using Mantel–Haenszel random‐effects models.^18^ We performed intention to treat (ITT) and per protocol (PP) analysis, where possible. Two researchers (MSF and AF) independently assessed the overall certainty of the evidence using the GRADE (Grading of Recommendations‐Assessment, Development and Evaluation) system (GRADEpro Guideline Development Tool 2015). Five factors were considered: 1) study limitations, 2) inconsistency, 3) imprecision, 4) indirectness, and 5) publication bias to determine the certainty of the evidence for each outcome. The certainty of the evidence was classified in four groups ranging from very low (any estimate of effect is very uncertain) through low and moderate to high (further research is very unlikely to change the confidence in estimates).

This report is adapted from a review initiated as part of the evidence syntheses for the *WHO guideline on health worker roles in safe abortion care and post‐abortion contraception*, which considered different cadres of non‐doctor providers separately.^12^ For the purpose of this paper, we synthesised research evidence for medical TOP provision by all cadres of non‐doctor providers compared with provision by doctors. This systematic review was conducted following the WHO principles for guideline development,^20^ and national experts in the field were consulted at WHO regional meetings. The PRISMA (preferred reporting items for systematic reviews and meta‐analyses) criteria were also considered.

### Funding

SS's and KGD's work with the present substudy was supported financially by the Swedish research council (ref. no. K213‐54X‐14212‐12‐5) and the Stockholm City County/Karolinska Institutet (ALF). BG and MD were supported by WHO. MSF was funded by the Norwegian Agency for Development Cooperation (NORAD). The funding bodies had no influence on study design or conducting the study.

## Results

### Search results

Our search yielded a total of 9425 citations, 8939 of which were unique. Studies were excluded after screening the title and abstract or the full‐text article (*n* = 60), based on study design, lacking a comparison group, or not assessing the outcomes of interest. Five reports from four RCTs,^21^, ^22^, ^23^, ^24^, ^25^ and one report of a prospective cohort study,^26^ met our inclusion criteria (Figure S1).

### Characteristics of the included studies

Three articles reported from low‐income settings, in Uganda and Nepal,^21^, ^22^, ^23^ one from a lower‐middle income setting, in India,^26^ one from a higher middle income setting, in Mexico,^24^ and one study was conducted in the high‐income setting of Sweden.^25^, ^27^ One RCT included ANMs and nurses,^21^ one RCT included nurse‐midwives (NMWs),^25^ and a third RCT included nurses,^24^ all compared with doctors. The prospective cohort study from India reported on the provision of medical abortion by Ayurvedic doctors (non‐conventional therapies doctors in the Indian system of medicine) or nurses, compared with doctors.^26^ The results from one RCT comparing midwife treatment of incomplete miscarriage with treatment by doctors were reported in two publications, covering effectiveness and safety,^22^ and satisfaction and acceptability outcomes,^23^ respectively.

The specific medical TOP or medical treatment of incomplete miscarriage regimens used differed across studies, but within each study all providers offered the same treatment regimens. Three RCTs,^21^, ^24^, ^25^ and one prospective cohort study,^26^ reported on treatment with combined mifepristone and misoprostol regimens for induced TOP with maximum gestational age of 63–70 days (mean 6.4–7.6 weeks of gestation). One RCT used 600 micrograms of oral misoprostol to treat incomplete miscarriage when uterine size was assessed to be below 12 weeks of gestation (mean 8.8 weeks of gestation) (Table 1).^22^, ^23^ A summary of outcome data is presented in Table S1.

**Table 1:** Characteristics of included studies

Author, location	Study design, period	Participatnts *n*, gestational age	Intervention	Indication and medical regimen	Outcome, method, and time of assessment
Warriner, 2011 Nepal Five district hospitals rural/peri‐urban area	RCT, equivalence trial Apr 2009–Mar 2010 ITT and PP analysis	1104 women (MLP *n* = 552, doctors *n* = 552) ≤63 days TOP	MLP *n* = 11 (nurses *n* = 8, Aux. NMW *n* = 3) Doctors *n* = 14 (ob/gyn *n *= 6; GP *n* = 3, BM/BS degree *n* = 5)	Induced TOP Mife: 200 milligram oral Miso 800 microgram vag Follow‐up: day 10–14	Primary: complete TOP Secondary: case management decisions Records of serious adverse events (blood transfusion, hospitalisation)
Kopp‐Kallner, 2014 Sweden Outpatient clinic	RCT, equivalence trial Feb 2011–Jul 2012 PP analysis Clinical trials reg 01612923 NCT	1180 women randomised (NMW *n* = 597; Doctor n=583) ≤63 days by LMP Mean GA 45 days in both groups TOP	NMW *n* = 2 experienced in MA and contraceptive counselling Doctors *n* = 34 with months to years of training and experience NMW theoretical and practical training in vaginal ultrasound Doctors no additional training Assigned provider medical history, clinical exam, ultrasound Intervention: Single NMW counselled, informed, examined, and treated woman Standard: doctor counselling and clinical examination, additional information and medication provided by NMW not in study	Induced TOP Day 1: 200 milligram mifepristone Oral Day 2–3: 800 microgram Misoprostol Vaginally or bucally at home or in clinic Repeat misoprostol 400 microgram oral if no bleeding at 3 hours after dose 1 Follow up: U‐Hcg after approx. 3 weeks	Primary outcome: efficacy successful completion of TOP without need for vacuum aspiration Secondary outcome: safety defined as no need for hospitalisation or blood transfusion and acceptability Complication need for causal treatment at an unscheduled visit up to 6 weeks after MA Efficacy and safety assessed by self‐administered questionnaires and medical records. Acceptability assessed through self‐administered questionnaires. Recorded need for second opinion and reason. Contaceptive method prior to and after MA
Olavarrieta, 2014 Mexico city Ministry of Health two government TOP clinics, one hospital	RCT, non‐inferiority trial Nov 2012–Jan 2013 Computer‐generated randomisation 14‐question acceptability survey with acceptability score. ITT and PP analysis	1017 women randomised (doctors *n* = 514; nurses *n* = 503); excluded nearly half for attempts at TOP prior to arrival GA <70 days Mean GA = 53 days (abdominal ultrasound) TOP	Nurses *n* = 7, doctors *n* = 8 No previous experience with MA/only managed MA under supervision Training 1.5 weeks for MA and 20 hours training in ultrasound Eligibility screening nurse participating in study Assigned provider: abdominal ultrasound for GA, gave instructions and counselling on post‐TOP contraception Follow‐up by assigned provider: clinical symtoms and abdominal ultrasound Satisfaction survey by study coordinator post TOP	Induced TOP Day 1: 200 milligram mifepristone oral Day 2: 800 microgram misoprostol buccal Follow‐up 7–15 days 800 microgram misoprostol if suspected continuing pregnancy or incomplete TOP. Then follow‐up after another 7–15 days	To assess effectiveness, safety and acceptability of nurses’ versus doctors provision of early medical TOP Complete TOP without surgical intervention. Checklist review of clinical symptoms and bleeding hx as well as results of abdominal ultrasound All adverse and serious adverse events were recorded 14‐item satisfaction survey Contraceptive counselling/provision
Klingberg‐Allvin, 2015 UGANDA Six primary health facilities in rural and peri‐urban regions	RCT, equivalence trial Mar 2013–Jul 2014 Computerised randomisation Per protocol analysis CONSORT guidelines Clinical trials NCT01844024	1010 randomised: midwife (*n* = 506); doctors (*n* = 504) PP analysis: 472 NMW and 483 doctors Mean GA 8.8 weeks Incomplete TOP	Midwives *n* = 29, doctors *n* = 13 Eligible participants worked at the maternal health section and involved in PAC; 5 days of training in PAC Assigned provider: detailed information on bleeding and pain and abnormal symptoms and importance of seeking care, and contraceptive counselling. RA: eligibility screening and enrolment, measured primary and secondary outcomes at follow‐up visit (midwives not in study)	Incomplete TOP Day 1: misoprostol 600 microgram oral, clinic analgesic (ibuprofen or paracetamol) and oral antibiotics according to national guidelines Follow up after 14–28 days	Primary outcome: complete TOP without need for surgical intervention within 14–28 days of initial treatment assessed through physical and pelvic exam Secondary outcomes: bleeding, pain, and unscheduled visits using symptom diary card and a visual analogue scale (VAS)
Cleeve et al. 2016 UGANDA Six primary health facilities in rural and peri‐urban regions	RCT, equivalence trial Analysis of secondary outcomes Mar 2013–Jul 2014 Computerised randomisation Clinical trials NCT01844024	1010 randomised: midwife (*n* = 506); doctors (*n* = 504) PP analysis: NMW = 472, doctors = 483 Mean GA 8.8 weeks Incomplete TOP	Midwives *n* = 29**,** doctors *n* = 13 Eligible participants worked at the maternal health section and involved in PAC; 5 days of training in PAC Assigned provider: detailed information on bleeding and pain and abnormal symptoms and importance of seeking care, and contraceptive counselling. RA: eligibility screening and enrolment, and measured primary and secondary outcomes at follow‐up visit (midwives not in study)	Incomplete TOP Misoprostol 600 microgram Oral (Day 1) with monitoring in clinic x4 hours Analgesics (ibuprofen or paracetamol) and oral antibiotics according to national guidelines	Primary outcome acceptability measured in expectations and satisfaction. Standardised questionnaires 14–28 days after treatment were used Overall acceptability was regarded as a dependent variable and measures such as bleeding, pain, feeling calm were regarded as independent variables reflecting the woman's treatment experience
Jejeeboy, 2012 INDIA five clinics operated by NGO in four urban areas	Prospective Cohort Dec 2008–May 2010 Providers rotated across sites and remained for around 6 weeks or 35–40 med ab. Clients unaware of which provider at which clinic Two‐sided equivalence design	1414 women assessed by pelvic exam 1225 eligible Ayurvedic (*n* = 404) Nurses (*n* = 416) Allopathic (*n* = 405) <8 weeks Recruited women lived within 1 hour of facility TOP	Allopathic doctors *n* = 10 Ayurvedic doctors *n* = 10 Nurses *n* = 10 No prior experiencein MA, BME, or assessed GA 10 days of training and field observation of a minimum of ten cases Provider gave information about own medical background, training, medical TOP procedure, side effects, post‐TOP contraception Verifier assess eligibility and TOP completeness, prescription of drugs, management of serious adverse events Exit interview by research coordinator recorded satisfaction	Induced TOP Medical eligibility determined by history and haemoglobin testing. GA determination by BME (by provider and verifier) and urine pregnancy test; no ultrasound used Day 1: 200 milligram mifepristone Oral, clinic Day 3: 400 microgram misoprostol Oral, clinic Follow‐up: day 15 and if needed on day 21 Routine antibiotics	Complete TOP measured as no need for subsequent surgical intervention (day 15–21). Complication rates, blood transfusion, and hospitalisation Secondary outcomes: correct eligibility assessment and assessment of complete TOP compared with verifier Acceptability: overall satisfaction with services and should they undergo procedure with same type of provider if needed. Recorded at exit interviews All provided post‐TOP contraception including referral for tubal ligation or IUD insertion

BME, bimanual exam; CRL, crown–rump length; GA, gestational age; GSD, gestational sac diameter; Hgb haemoglobin level; IIT, intention to treat; LARC, long‐acting reversible contraception; LMP, last menstrual period; MA, medical abortion; MLP, mid‐level provider; NMW, nurse midwife; PAC, post‐abortion care; PP, per protocol; RA, research assistant; TOP, termination of pregnancy.

### Outcome measures

#### Effectiveness

All four included RCTs, and one prospective cohort study,^26^ defined effectiveness of medical TOP as complete TOP without the need for vacuum aspiration. Clinical assessment was the primary method for determining TOP completion. In addition, one RCT reported on routine use of low‐sensitivity urine pregnancy testing,^25^ and one study used abdominal ultrasound to confirm gestational length and TOP completion.^24^

**Figure 1:**
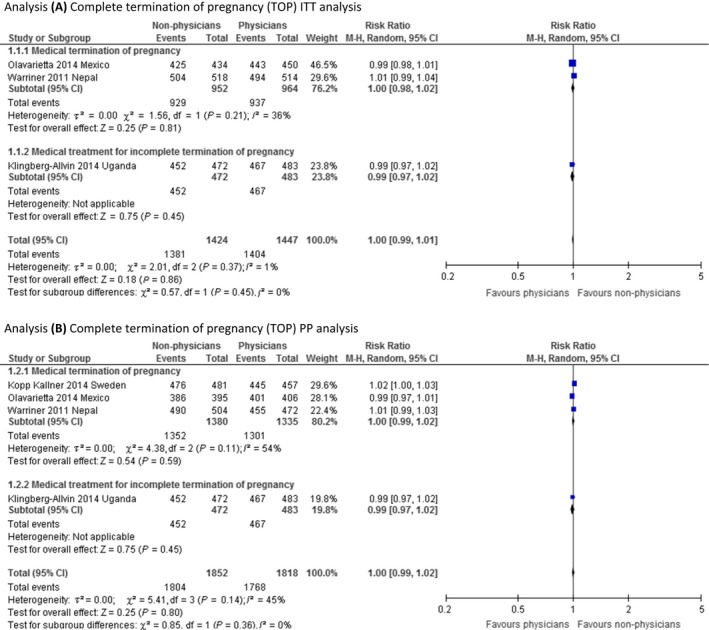
Meta‐analysis of effectiveness.

In our ITT analysis for the outcome of effectiveness, three RCTs provided enough information to be included in the analysis.^21^, ^22^, ^24^ Even though Klingberg‐Allvin et al.^22^ reported results PP, we consider the same population number in both the ITT and PP analysis from this study, as the population was only marginally different in the two groups. Our meta‐analysis show that effectiveness, measured as the complete TOP rate, is probably equivalent between the provider groups, as the effect estimate (RR 1.00, 95% CI 0.99–1.01) show no clinically significant difference with very narrow confidence intervals (Figure 1, analysis A). Subgroup analyses shows that complete TOP rates may be similar between provider groups whether women are seeking medical TOP or medical treatment of incomplete TOP. The validity of the evidence is moderate (Table 2). These findings were verified by the PP analysis carried out with all four RCTs reporting on this outcome (RR 1.00, 95% CI 0.99–1.02) (Figure 1, analysis B).

**Table 2:** Certainty of evidence (GRADE)

Non‐doctors compared with doctors providing medical termination of pregnancy						
**Patient or population:** pregnant women seeking medical termination of pregnancy						
**Intervention:** non‐doctors						
**Comparison:** doctors						
Outcomes	Anticipated absolute effects^a^ (95% CI)		Relative effect (95% CI)	No. of participants (studies)	Quality of the evidence (GRADE)	Comments
	Risk with doctors	Risk with non‐doctors				
**Effectiveness** Complete TOP without surgical intervention ITT analysis	970 per 1 000	**970 per 1000** (961–980)	**RR 1.00** (0.99–1.01)	2871 (3 RCTs)	⨁⨁⨁◯ MODERATE^b^	**Interpretation:** There is probably little or no difference in the effectiveness (complete TOP without surgical intervention) of medical termination of pregnancy among women treated and followed up by non‐ doctors versus doctors
**Effectiveness** Complete TOP without surgical intervention PP analysis	972 per 1 000	**972 per 1000** (963–992)	**RR 1.00** (0.99–1.02)	3670 (4 RCTs)	⨁⨁⨁◯ MODERATE^b^	**Interpretation:** There is probably little or no difference in the effectiveness (complete TOP without surgical intervention) of medical termination of pregnancy among women treated and followed up by non‐ doctors versus doctors
**Safety** Serious adverse events (need for hospital admission, blood transfusion, or death)	1 per 1 000	**0 per 1000** (0–0)	not estimable	2915 (3 studies)	–	**Interpretation:** We are uncertain of the effect estimate because there are very few serious events and we can thus not generalise about how different or similar the effect of being treated and followed up by non‐doctors versus doctors is
**Acceptability/satisfaction** Would you recommend to a friend? ITT analysis	985 per 1 000	**985 per 1000** (975–994)	**RR 1.00** (0.99–1.01)	1842 (2 RCTs)	⨁⨁⨁◯ MODERATE^b^	**Interpretation:** There is probably little or no difference in the acceptability/satisfaction (would recommend to a friend) of medical termination of pregnancy among women treated and followed up by non‐doctors versus doctors
**Acceptability/satisfaction** Would you recommend to a friend? PP analysis	797 per 1 000	**948 per 1000** (406–1 000)	**RR 1.19** (0.51–2.75)	2021 (2 RCTs)	⨁◯◯◯ VERY LOW^b^^,^ c ^,^ d	**Interpretation:** We are uncertain of the effect estimate because the validity of the evidence has been assed as very low
**Acceptability/satisfaction** Overall satisfaction ITT analysis	969 per 1 000	**969 per 1000** (969–979)	**RR 1.00** (1.00–1.01)	1838 (2 RCTs)	⨁⨁⨁◯ MODERATE^b^	**Interpretation:** There is probably little or no difference in the acceptability/satisfaction (overall satisfaction) of medical termination of pregnancy among women treated and followed up by non‐doctors versus doctors
**GRADE working group grades of evidence**						
**High quality:** we are very confident that the true effect lies close to that of the estimate of the effect						
**Moderate quality:** we are moderately confident in the effect estimate – the true effect is likely to be close to the estimate of the effect, but there is a possibility that it is substantially different						
**Low quality**: our confidence in the effect estimate is limited – the true effect may be substantially different from the estimate of the effect						
**Very low quality:** we have very little confidence in the effect estimate – the true effect is likely to be substantially different from the estimate of effect.						

a The risk in the intervention group (and its 95% CI) is based on the assumed risk in the comparison group and the relative effect of the intervention (and its 95% CI).

b Overall unclear risk of bias in included studies.

c Very high heterogeneity.

d Somewhat broad 95% CI that crosses the line of no effect.

#### Safety

Across all studies, only one serious adverse event was recorded: one woman was hospitalised for heavy bleeding and underwent surgical TOP without further complications (Table S1).^24^ As a result of rarity of recorded events, meta‐analysis and assessment of validity of the evidence was not performed; studies were not powered to detect differences in safety according to provider. Only one RCT clearly defined safety outcomes in the trial: need for hospitalisation or blood transfusion.^25^ Another RCT stated that all adverse and serious adverse events were recorded and analysed to allow for safety reporting, but did not specify which events were considered.^24^ The prospective cohort study defined serious adverse events as haemorrhage requiring blood transfusion and/or need for hospitalisation, but did not clearly state what measures were considered when reporting on safety. This study reported that no women had serious complications or required blood transfusion or hospitalisation.^26^ The rest of the RCTs did not make a clear definition of serious adverse events.

#### Acceptability

**Figure 2:**
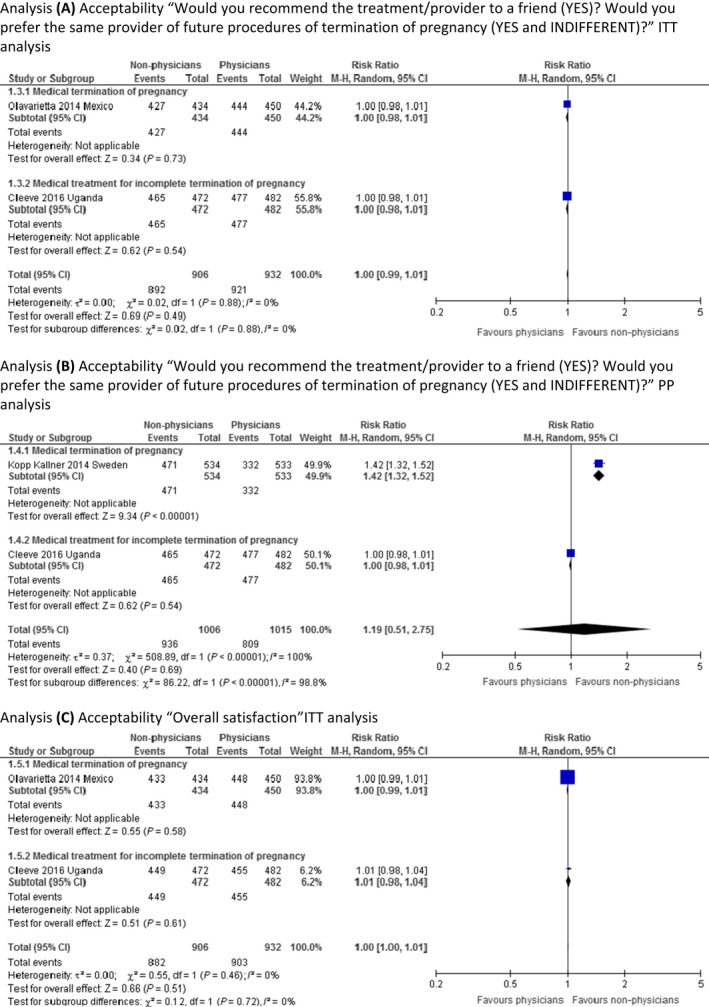
Meta‐analysis of acceptability.

Three RCTs provided enough information to be included in the ITT analyses for the outcome of acceptability/satisfaction with treatment and/or service outcomes.^23^, ^24^, ^25^ Cleeve et al.^23^ reported PP analysis only, but we report the same population number in both ITT and PP analyses from this study because the population was only marginally different in the two cases. The meta‐analyses report on women's acceptance of the allocated treatment and/or provider in terms of whether women would recommend the same treatment or provider to a friend, or whether they would prefer the same type of provider in case of a future TOP. These statements were considered to similarly assess the woman's acceptance of the procedure/provider, as it is unlikely that you would recommend a treatment to a friend that you would not undergo yourself. Our meta‐analysis show that the result is probably equivalent between provider groups, as the effect estimate shows no clinically significant difference, with a narrow confidence interval (RR 1.00, 95% CI 0.99–1.01; Figure 2, analysis A). The validity of the evidence is moderate (Table 2). We are uncertain if the PP analysis can verify the findings by the ITT analysis for this outcome because the validity of the evidence was assessed as very low (Figure 2, analysis B).

Two RCTs reported on overall satisfaction.^23^, ^24^ Our ITT meta‐analysis show that women are probably equally satisfied ‘all in all’ with the provider, regardless of provider allocation (RR 1.00, 95% CI 1.00–1.01; Figure 2, analysis C). The validity of evidence is moderate (Table 2). As there was only one study with enough information provided,^23^ we did not perform a PP analysis for this outcome, also this study was already included in the ITT analysis and the population was only marginally different in the two cases of analyses provided by the study authors.

The prospective cohort study also reported on satisfaction. At recruitment, a 0.5% refusal rate of the assigned provider was registered; however, at the time of exit interviews satisfaction with the assigned provider was high (98–99%). Almost all women in this study stated that they would undergo treatment from the same provider type again.^26^ Two articles did not report any outcomes on acceptability or satisfaction.^21^, ^22^

## Discussion

### Quality of evidence

An assessment of the risk of bias in the RCTs showed that there was no selection bias (sequence generation and allocation concealment) in the included RCTs. There was risk of performance bias as well as detection bias in all studies, as participants were not blinded to provider type, and there was no report of the blinding of outcome assessors. Attrition bias, or incomplete outcome data, was adequately addressed in four reports (three RCTs).^21^, ^22^, ^23^, ^24^ Whether there was selective reporting bias (analyses with statistically significant results are more likely to be reported than non‐significant results) was unclear for all RCTs (Table S2A). Risk of bias in the included prospective cohort study was found to be low (Table S2B).^26^

The validity of the evidence was assessed using the GRADE tool and ranged from very low to moderate (Table 2).^28^ For the outcomes where we assessed the quality of evidence as moderate, the main reason for downgrading was the unclear risk of bias in the included studies. The downgrading of the outcome where the quality of evidence was assessed as very low was for unclear risk of bias of included studies, very high heterogeneity, and imprecision (wide 95% CI of the effect estimate that crossed the line of no effect).

### Main findings

We conclude that medical TOP and medical treatment of incomplete miscarriage performed by trained non‐doctor providers in the first trimester is probably as effective as treatment provided by doctors. We also conclude that women are probably equally satisfied with their provider regardless of who treats or manages their medical TOP.

### Strengths and limitations

Despite the limited number of included studies, this is the largest systematic review of the provision of first‐trimester medical TOP by non‐doctor providers (ANMs, nurses, midwives, and non‐conventional therapies doctors), compared with doctors, and also the first to include the medical management of incomplete miscarriage. In addition, this is the first systematic review to report on women's acceptance of provider and services, and women's overall acceptance in terms of satisfaction with medical treatment for early TOP and incomplete miscarriage.

The generalisability of our findings may be low. Included studies were conducted in diverse settings and evaluated treatment of different populations of women (total *n* = 5823) from Nepal, Sweden, Mexico, Uganda, and India. The number of included providers varied between studies (non‐doctors, *n* = 69; doctors, *n* = 79), and the variability in training and prior professional experience was high. Although medical TOP is considered a relatively safe procedure, the included studies were not powered to report on significant differences regarding serious adverse events. We can therefore not conclude whether the safety is equal, higher, or lower between the two groups of medical TOP providers. Also, the definitions of adverse events, serious adverse events, and the measurement of safety were unclear in several studies.

### Interpretation

Our findings on effectiveness, defined as complete TOP without surgical intervention, when non‐doctors provided medical TOP are consistent with findings of previously published systematic reviews.^9^, ^10^, ^11^ The previous reviews conclude that the provision of medical TOP and medical treatment for incomplete miscarriage is as effective and safe when provided by non‐doctors as when provided by doctors; however, these reviews were broader in scope and evaluated non‐doctor provision of medical TOP in addition to surgical TOP. Our review is more comprehensive in its focus on the provision of medical TOP and medical treatment of incomplete miscarriage, including women's acceptance of the provider and services, and more and newer studies are included.

We defined the safety outcome as serious adverse events and concluded that the included studies were not powered to detect serious adverse events. Although all included studies reported on safety, the definitions and distinctions between serious adverse events, adverse events, and minor common complications were not clear in several studies. Although more events need to be reported to make a robust statistical generalisation about the influence of provider type on the likelihood of serious adverse events, the rarity of these events overall is very reassuring. Medical TOP in the first trimester using mifepristone and misoprostol is well established as an effective and safe method for TOP, and is feasible in different settings,^13^ with a number of previous studies reporting high rates of complete TOP and very few serious adverse events.^29^ We also conclude that standardised approaches to defining and reporting serious adverse events in subsequent studies would further strengthen our conclusions.

We found that women's acceptance of treatment and management of medical TOP or incomplete miscarriage is probably equally high between those provided by doctors versus non‐doctors. Furthermore, women probably equally prefer the same provider if they should need another TOP. Few previous studies and no systematic reviews have examined women's satisfaction with procedure and provider when seeking treatment for TOP and incomplete miscarriage. If women find the available procedures and providers acceptable, the likelihood that they will seek safe TOP providers when needed increases.^8^

We hypothesise that if we increase the numbers of facilities where non‐doctors are able to provide medical treatment for TOP, this can increase access to safe TOP and medical treatment of incomplete miscarriage. Provision by non‐doctor providers in a variety of facility‐based settings as part of routine care does not only increase access to care, but also helps de‐medicalise and normalise TOP services, which reduces stigma and augments women's care seeking, thereby preventing any delay to treatment.

## Conclusion

In this systematic review, we establish further support for non‐doctor provision of medical TOP and medical treatment of incomplete miscarriage. The effectiveness of treatment is probably similar regardless of whether it is provided by a non‐doctor provider or by a doctor (moderate validity of the evidence). Moreover, women's overall acceptance of their provider is probably similar regardless of allocation (very low to moderate validity of the evidence). The effect on safety is uncertain as our outcome measure, serious adverse event, is rare and the studies are not powered to allow for a generalisation about the effect treatment by the non‐doctors versus doctors have on safety. These findings are important for the successful implementation of non‐doctor provision of TOP care, and stresses the necessity to scale‐up such provision as soon as possible to increase access to safe TOP care.

### Disclosure of interests

None declared. Completed disclosure of interests form available to view online as supporting Information.

### Contribution to authorship

The initial review was conducted as part of the WHO guideline development for health worker roles. BG had overall responsibility of the guideline development and coordinated the work. KGD initiated the present substudy. SS and MD conducted the initial search, and independently screened the retrieved citations, abstracts, and full texts when necessary. They also systemised the evidence using standard abstraction forms. MSF carried out the analysis and assessed the overall quality and validity of the evidence with the GRADE (grading of recommendations assessment, development and evaluation) system (with her co‐worker). SS and MD wrote the first draft of the manuscript. All authors participated in the revision and writing of the final manuscript.

### Details of ethics approval

Not required.

## Supporting information


**Figure S1.** PRISMA flowchart.

**Table S1.** Summary of outcome data RCTs.

**Table S2.** A, risk of bias assessment for included RCTs; B, risk of bias assessment for prospective cohort studies (Medical TOP and facility‐based providers).

**Appendix S1.** Search strategy.
