# Supplementary material for: Effectiveness, safety, and acceptability of first‐trimester medical termination of pregnancy performed by non‐doctor providers: a systematic review
Source: BJOG. 2017 Aug 17;124(13):1928–40. doi: 10.1111/1471-0528.14712 (PMC5724486; doi:10.1111/1471-0528.14712)
Supplement: Supplementary file 1 — Figure S1. PRISMA flowchart. [file BJO-124-1928-s001.pdf]

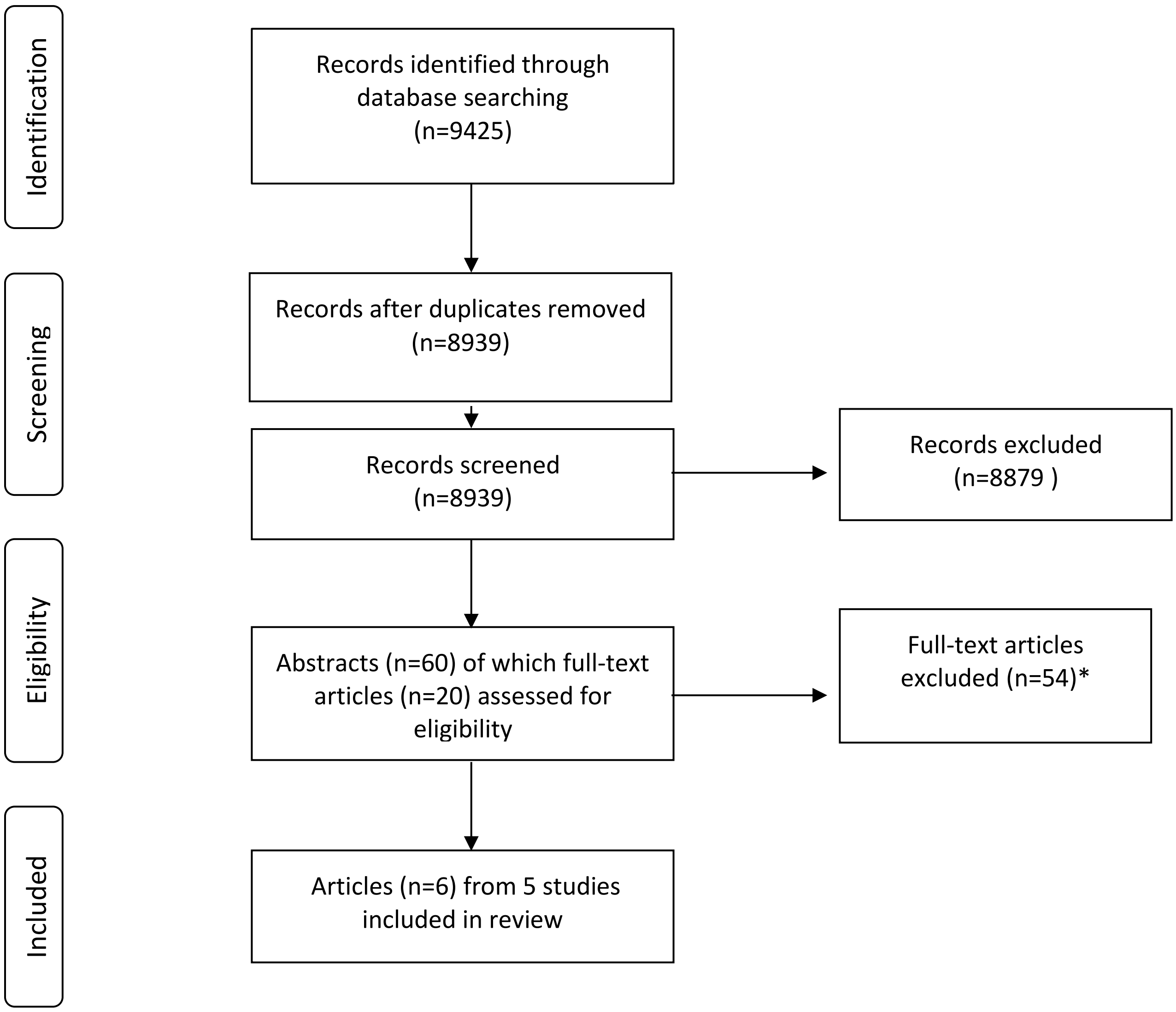

*Reasons for exclusion: No clinical trial registration (n=1), methods not included (n=3), lack of comparison group (n=8), not assessing outcomes of interest (n=42)

Figure S1. PRISMA flowchart
