# Supplementary material for: Effectiveness, safety, and acceptability of first‐trimester medical termination of pregnancy performed by non‐doctor providers: a systematic review
Source: BJOG. 2017 Aug 17;124(13):1928–40. doi: 10.1111/1471-0528.14712 (PMC5724486; doi:10.1111/1471-0528.14712)
Supplement: Supplementary file 2 — Table S1. Summary of outcome data RCTs. [file BJO-124-1928-s002.pdf]

Table S1. Summary of outcome data RCTs

Study, year, country, design	Non-physician	Physician	Risk ratio M-H, Random (95 % CI)
Outcome 1. Effectiveness - complete TOP (no need for surgical interventions)			
Klingberg-Allvin, 2014, Uganda, e-RCT	29 MW<br>PP: 452/472	13 P<br>PP: 467/483	MW group vs P group<br>PP*: 0.99 (0.97, 1.02)
Kopp Kallner, 2014, Sweden, e-RCT	2 NMW<br>ITT: *Not available*<br>PP: 476/481	34 P<br>ITT: *Not available*<br>PP: 445/457	NMW group vs P group<br>ITT: *Not available*<br>PP: 1.02 (1.00, 1.03)
Olavarietta, 2014, Mexico, ni-RCT	7 N<br>ITT: 425/434<br>PP: 386/395	8 P<br>ITT: 443/450<br>PP: 401/406	N group vs P group<br>ITT: 0.99 (0.98, 1.01)<br>PP: 0.99 (0.97, 1.01)
Warriner, 2011, Nepal, e-RCT	8 N + 3 ANM<br>ITT: 504 /518<br>PP: 490/504	14 P<br>ITT: 494/514<br>PP: 455/472	N+ANM group vs P group<br>ITT: 1.01 (0.99, 1.04)<br>PP: 1.01 (0.99, 1.03)
Outcome 2. Safety - serious adverse events (blood transfusion, hospitalization, deaths)			
Klingberg-Allvin, 2014, Uganda, e-RCT	29 MW<br>PP: 0/472	13 P<br>PP: 0/483	MW group vs P group<br>PP: *Not estimable*
Kopp Kallner, 2014, Sweden, e-RCT	2 NMW<br>ITT: *Not available* PP: 0/473	34 P<br>ITT: *Not available*<br>PP: 0/443	NMW group vs P group<br>ITT: *Not available*<br>PP: *Not estimable*
Olavarietta, 2014, Mexico, ni-RCT	7N<br>ITT: 0/434<br>PP: 0/395	8 P<br>ITT: 1/450<br>PP: 0/406	N group vs P group<br>ITT: *Not estimable*<br>PP: *Not estimable*
Warriner, 2011, Nepal, e-RCT	8 N + 3 ANM<br>ITT: 0 /518<br>PP: 0/504	14 P<br>ITT: 0/514<br>PP: 0/472	N+ ANM group vs P group<br>ITT: *Not estimable*<br>PP: *Not estimable*
Outcome 3. Acceptability – satisfaction with provider<br>A. Would you recommend the treatment/provider to a friend (YES)? Would you prefer the same provider of future procedures of termination of pregnancy (YES and INDIFFERENT)?			
Cleeve, 2016, Uganda, e-RCT	29 MW<br>PP: 465 /472	13 P<br>PP: 477/482	MW group vs P group<br>PP*: 1.00 (0.98, 1.01)
Kopp Kallner, 2014, Sweden, e-RCT	2 NMW<br>ITT: 471/534	34 P<br>ITT: 332/533	NMW group vs P group<br>ITT: 1.42 (1.32, 1.52)
Olavarietta, 2014, Mexico, ni-RCT	7N<br>ITT: 427/434	8 P<br>ITT: 444/450	N group vs P group<br>ITT: 1.00 (0.98, 1.01)
B. How satisfied are you with the provider (SATISFIED and VERY SATISFIED)? Overall acceptability (SATISFACTORY).			
Cleeve, 2016, Uganda, e-RCT	29 MW<br>PP: 449/472	13 P<br>PP: 455/482	MW group vs P group<br>PP: 1.01 (0.98, 1.04)
Olavarietta, 2014, Mexico, ni-RCT	7N<br>ITT: 433/434	8 P<br>ITT: 448/450	N group vs P group<br>ITT: 1.00 (0.99, 1.01)

* The population is marginally different between PP and ITT thus the same population used in both the PP and ITT analysis

Abbreviations 95% CI 95 % confidence interval; e-RCT, equivalence randomized controlled trial; ITT, intention-to-treat analysis; ANM, auxiliary nurse midwives; M-H, Mantel-Haenszel random-effects models; MW, midwives, N, nurses; NMW, nurse midwives; ni-RCT; non-inferiority randomized controlled trial; P, physicians; PP, per protocol analysis ; TOP, termination of pregnancy
