# Supplementary material for: Effectiveness, safety, and acceptability of first‐trimester medical termination of pregnancy performed by non‐doctor providers: a systematic review
Source: BJOG. 2017 Aug 17;124(13):1928–40. doi: 10.1111/1471-0528.14712 (PMC5724486; doi:10.1111/1471-0528.14712)
Supplement: Supplementary file 3 — Table S2. A, risk of bias assessment for included RCTs; B, risk of bias assessment for prospective cohort studies (Medical TOP and facility‐based providers). [file BJO-124-1928-s003.pdf]

Table S2A. Risk of bias assessment for included RCTs

AUTHOR, DATE,	SEQUENCE GENERATION SELECTION BIAS	ALLOCATION CONCEALMENT	BLINDING OF PARTICIPANTS	INCOMPLETE OUTCOME DATA	SELECTIVE OUTCOME REPORTING	OTHER SOURCES OF BIAS	OVERALL RISK OF BIAS
**WARRINER, 2011**	Adequate: Y<br><br>Computer-generated randomization in blocks of six	Adequate: Y<br><br>Sequentially numbered, sealed opaque envelopes	Adequate: N<br><br>No blinding to provider type<br>Separate exam rooms and waiting areas for different provider types	Adequately addressed: Y<br><br>4% lost to follow-up balanced in numbers across intervention groups, reasons for missing data similar<br>ITT and PP analyses performed	Free of selective reporting: Unclear	Free of other bias: N<br><br>Small number of providers varying professional experience; in multivariate analysis, years of experience did not have impact<br>No independent verification of clinical assessments<br>Mean number of women treated by individual provider unclear<br>Nurses and Aux Nurse Midwives with different training backgrounds and years of experience lumped together	Unclear risk of bias
**KOPP KALLNER, 2014**	Adequate: Y<br><br>Computer generated randomization in blocks of 10	Adequate: Y<br><br>Sequentially numbered, sealed opaque envelopes	Adequate: N<br><br>No blinding to provider type	Adequately addressed: N<br><br>12% lost to follow-up similar in both arms<br><br>PP analysis	Free of selective reporting: Unclear	Free of other bias: N<br><br>Varying levels of professional experience/training among provider groups<br>No independent verification of clinical assessments<br>Mean number of women treated by individual provider unclear<br>Small number of providers to evaluate intervention	Unclear risk of bias
**OLAVARRIETA,2014**	Adequate: Y<br><br>Computer generated randomization	Adequate: Y<br><br>Sequentially numbered, sealed opaque envelopes	Adequate: N<br><br>No blinding to provider type	Adequately addressed:<br><br>> 10% lost to follow-up, similar in both arms<br>ITT and PP analyses performed	Free of selective reporting: Unclear	Free of other bias: N<br><br>Varying levels of professional experience/training among provider groups<br>Mean number of women treated by individual provider unclear<br>No independent verification of clinical assessments<br>Small number of providers to evaluate intervention	Unclear risk of bias
**KLINGBERG-ALLVIN, 2014**	Adequate: Y<br><br>Computer generated randomization in blocks of 12	Adequate: Y<br><br>Sequentially numbered, sealed opaque envelopes	Adequate: N<br><br>No blinding to provider type	Adequately addressed: Y<br><br>5 and 3 % lost to follow-up<br>PP analyses reported as noted only 2 protocol violations in ITT population	Free of selective reporting: Unclear	Free of other bias: N<br><br>Varying levels of professional experience/training among provider groups<br>No independent verification of clinical assessments<br>Mean number of women treated by individual provider unclear<br>Small number of providers to evaluate intervention	Unclear risk of bias
**CLEEVE 2016**<br><br>**SECONDARY OUTCOMES FROM RCT-EQUIVALENCE TRIAL**	Adequate: Y<br><br>Computer generated randomization in blocks of 12	Adequate: Y<br><br>Sequentially numbered, sealed opaque envelopes	Adequate: N<br><br>No blinding to provider type	Adequately addressed: Y<br><br>5 and 3 % lost to follow-up<br>PP analyses reported as noted only 2 protocol violations in ITT population	Free of selective reporting: Unclear	Free of other bias: N<br><br>Varying levels of professional experience/training among provider groups<br>No independent verification of clinical assessments<br>Mean number of women treated by individual provider unclear<br>Small number of providers to evaluate intervention	Unclear risk of bias

Abbreviations: ITT intention to treat, PP per prococol

Table S2B. Risk of bias assessment for prospective –cohort studies (Medical TOP and facility-based providers)

AUTHOR, DATE STUDY DESIGN	EXPOSED/ UNEXPOSED FROM SAME POPULATION?	CONFIDENT IN EXPOSURE ASSESSMENT ?	CONFIDENT THAT OUTCOME OF INTEREST NOT PRESENT AT START OF STUDY?	ADEQUATE MATCHING OR ADJUSTMENT FOR VARIABLES ASSOCIATED WITH OUTCOME OF INTEREST?	CONFIDENT IN ASSESSMENT OF PRESENCE/ABSENCE OF PROGNOSTIC FACTORS?	CONFIDENT IN ASSESSMENT OF OUTCOME?	ADEQUATE FOLLOW-UP?	SIMILAR CO-INTERVENTIONS ACROSS GROUPS?	RISK OF BIAS
**JEJEEBHOY, 2012 PROSPECTIVE COHORT**	Yes<br>All women seeking medical TOP	Yes	No<br>No providers had experience of medical abortion and completed same 10-d training course<br>Mean number of procedures per provider unclear	Probably Yes<br>Similar baseline characteristics of women treated<br>No statement of mean GA of women treated by each provider type	Probably Yes<br>Same regimen for medical TOP	Probably Yes<br>Clinical assessments of eligibility and complete TOP verified by certified provider	Yes<br>5% loss to follow-up	Yes	Low/unclear
