# Supplementary material for: Effectiveness, safety, and acceptability of first‐trimester medical termination of pregnancy performed by non‐doctor providers: a systematic review
Source: BJOG. 2017 Aug 17;124(13):1928–40. doi: 10.1111/1471-0528.14712 (PMC5724486; doi:10.1111/1471-0528.14712)
Supplement: Supplementary file 4 — Appendix S1. Search strategy. [file BJO-124-1928-s004.pdf]

Appendix S1. Search strategy

PubMed

#	Search Terms
**1**	**<u>Non-specialty physicians</u>**<br>("Physicians, Primary Care"[Mesh] OR "General Practitioners"[Mesh]) OR "Physicians, Family"[Mesh] OR “Clinical officer” [tiab] OR “Non-specialist doctors” [tiab])
**2**	**<u>Non-allopathic physician</u>**<br>("Health Services, Indigenous"[Mesh] OR "Medicine, Traditional"[Mesh] OR "Integrative Medicine"[Mesh] OR "Complementary Therapies"[Mesh] OR "Herbal Medicine"[Mesh] OR "Osteopathic medicine"[MeSH Terms] OR "Chiropractic"[Mesh] OR "Naturopathy"[Mesh] OR "Medicine, Ayurvedic"[Mesh] OR "Medicine, Chinese Traditional"[Mesh] OR “complementary medicine” [tiab] OR “alternative medicine”[tiab] OR “chiropractic”[tiab] OR “osteopathic medicine”[tiab] OR “naturopathy”[tiab] OR “traditional medicine”[tiab] OR “ayurveda”[tiab] OR “Chinese medicine”[tiab] OR “TCM”[tiab]) AND ("Health Personnel"[Mesh] OR “personnel” [tiab] OR “provider”[tiab] OR “practitioner” [tiab] OR “professional” [tiab] OR “clinician”[tiab] OR “physician” [tiab])
**3**	**<u>Advanced level associate clinician and associate clinicians</u>**<br>“Assistant medical officer” [tiab] OR “clinical officer” [tiab] OR “medical licentiate” [tiab] OR “health officer” [tiab] OR “physician assistant” [tiab] OR “surgical technician” [tiab] OR “medical technician” [tiab] OR “non-physician clinician” [tiab] OR “Allied Health Personnel"[Mesh] OR "Hospital Auxiliaries"[Mesh] OR “Physician Assistants"[Mesh] OR "Nurse Clinicians"[Mesh]) OR "Nurse Practitioners"[Mesh]) OR “advanced practice nurse*” [tiab] OR “nurse practitioner*” [tiab] OR “Clinical officer” [tiab] OR “Medical assistant” [tiab] OR “health officer” [tiab] OR “clinical associate” [tiab] OR “physician assistant” [tiab] OR “clinical nurse specialist*” [tiab] OR Non-physician clinician* [tiab] OR “non-professional clinician” [tiab]
**4**	**<u>Midwives</u>**<br>“Midwifery” [MeSH] OR “midwifery” [tiab] OR “midwives” [tiab] OR “Registered midwife” [tiab] OR “midwife” [tiab] OR “community midwife” [tiab] OR “Nurse Midwife” [Mesh] OR “CNM”[tiab] OR “Certified nurse midwife”[tiab]
**5**	**<u>Nurses</u>**<br>Nurse [MeSH] OR “nurse*” [tiab] OR “Nursing Personnel” [tiab] OR “Registered nurse*” [tiab] OR “RN”[tiab] OR “practice nurse*” [tiab] OR “licensed nurse” [tiab] OR “diploma nurse” [tiab] OR “BS nurse*” [tiab] OR “nurse clinician*” [tiab] OR "Nurses, International"[Mesh] OR "Nurses, Community Health"[Mesh] OR "Nurses, Public Health"[Mesh] OR “nurse clinician*” [tiab] OR “LPN”[tiab] OR “ LP nurse”[tiab]
**6**	**<u>Auxiliary nurses and midwives</u>**<br>Nurses' Aides [MeSH] OR nurses' aides [tiab] OR nurses aide [tiab] OR Nurses Aides [tiab] OR Nurses' Aide [tiab] OR nurse aide* [tiab] OR Nursing Auxiliaries [tiab] OR Nursing Auxiliary [tiab] OR Auxiliary nurse* [tiab] OR auxiliary nurse midwife [tiab] OR auxiliary nurse midwives [tiab] OR auxiliary midwife [tiab] OR auxiliary midwives [tiab] OR nurse assistant [tiab]
**7**	#1 OR #2 OR #3 OR #4 OR #5 OR #6
**8**	"Abortion, Induced"[Mesh] OR "Abortion, Incomplete"[Mesh] OR "Abortion, Spontaneous"[Mesh] OR “abortion” [tiab] OR “miscarriage”[tiab] OR “pregnancy termination”[tiab] OR “termination of pregnancy”[tiab] OR “postabortion care” [tiab] OR “incomplete abortion”[tiab] OR "Mifepristone"[Mesh] OR "Misoprostol"[Mesh] OR “RU486”[tiab] OR “mifegyne”[tiab] OR “Cytotec”[tiab] OR “Medabon”[tiab] OR “medication abortion”[tiab] OR “medical abortion”[tiab]

**9**	#7 AND #8
**10**	"Pregnancy"[Mesh] OR "Pregnancy, Unplanned"[Mesh] OR “pregnancy” [tiab] OR “IUP” [tiab]
**11**	#8 OR #10
**12**	#7 AND #11
**13**	("Eligibility Determination"[Mesh] OR "Ultrasonography"[Mesh] OR "Pregnancy Tests"[Mesh] OR "Checklist"[Mesh] OR "Medical History Taking"[Mesh] OR "Physical Examination"[Mesh] OR “eligibility”[tiab] OR “eligibility assessment”[tiab] OR “pregnancy dating”[tiab] OR “gestational age”[tiab] OR “pregnancy test”[tiab] OR “checklist”[tiab] OR “medical history”[tiab] OR “bimanual examination”[tiab] OR “ultrasound”[tiab] OR “ultrasonography”[tiab] OR “sonogram”[tiab] OR “last menstrual period”[tiab] OR “LMP”[tiab]
**14**	#12 AND #13
**15**	"Nausea"[Mesh] OR "Fever"[Mesh] OR "Diarrhea"[Mesh] OR "Chills"[Mesh] OR "Pain"[Mesh] OR "Acute Pain"[Mesh] OR "Pain Management"[Mesh] OR “nausea” [tiab] OR “fever”[tiab] OR “diarrhea”[tiab] OR “diarrhoea”[tiab] OR “chills”[tiab] OR “pain”[tiab]
**16**	#12 AND #15
**17**	"Hemorrhage"[Mesh]) OR "Postoperative Hemorrhage"[Mesh] OR "Uterine Hemorrhage"[Mesh] OR "Postpartum Hemorrhage"[Mesh] OR "Infection"[Mesh] OR "Pelvic Infection"[Mesh] OR "Uterine Perforation"[Mesh] OR "Uterine Rupture"[Mesh] OR "Pregnancy Complications"[Mesh] OR "Postoperative Complications"[Mesh] OR "Intraoperative Complications"[Mesh] OR "Emergency Treatment"[Mesh] OR “Abortion, Septic” [Mesh] OR “haemorrhage” [tiab] OR “haemorrhage”[tiab] OR “hemorrhage”[tiab] OR “bleeding”[tiab] OR “endometritis”[tiab] OR “parametritis”[tiab] OR “metritis” [tiab] OR “pelvic infection” [tiab] OR “uterine infection” [tiab] OR “uterine perforation” [tiab] OR “abortion-related complications” [tiab] OR “emergency care” [tiab] OR “ongoing pregnancy” [tiab] OR “ectopic pregnancy” [tiab] OR “emergency treatment” [tiab] OR “EmOC”[tiab] OR “emergency obstetric care”[tiab] OR “complications”[tiab]
**18**	#12 AND #17
**16**	#9 OR #14 OR #16 OR #18

Global Index Medicus

**#**	**Search terms**	**Database**
1	("abortion" or "miscarriage" or "incomplete abortion" or “postabortion care”) AND (“misoprostol” or “medical” or “medication”)	Total
		LILACS
		WPRIM
		IMEMR
		IMSEAR
		WHOLIS
		AIM

EMBASE

**#**	**Search terms**
**1**	'general practitioner'/exp OR ‘general practitioner’:ab,ti OR ‘family physician’:ab,ti OR ‘clinical officer’:ab,ti OR 'nonspecialist doctor,:ab,ti
**2**	'alternative medicine'/exp OR 'alternative medicine' OR 'integrative medicine'/exp OR 'integrative medicine' OR 'complementary medicine':ab,ti OR 'osteopathic medicine'/exp OR

	'osteopathic medicine' OR ('chinese medicine'/exp OR 'chinese medicine') OR 'ayurveda'/exp OR 'ayurveda' OR ('chiropractic'/exp OR 'chiropractic') OR 'osteopathic medicine':ab,ti OR 'chinese medicine':ab,ti OR 'ayurveda':ab,ti OR 'chiropractic'/exp OR 'chiropractic' OR 'traditional medicine'/exp OR 'traditional medicine' OR 'integrative medicine':ab,ti OR 'naturopathy':ab,ti
**3**	'health care personnel'/exp OR 'health practitioner'/exp OR 'provider':ab,ti OR 'practitioner':ab,ti OR 'physician':ab,ti OR 'professional':ab,ti OR 'clinician':ab,ti
**4**	#2 AND #3
**5**	'physician assistant'/exp OR 'nurse practitioner'/exp OR 'paramedical personnel'/exp OR 'hospital personnel'/exp OR 'assistant medical officer':ab,ti OR 'clinical officer':ab,ti OR 'physician assistant':ab,ti OR 'assistant medical officer':ab,ti OR 'medical licentiate':ab,ti OR 'health officer':ab,ti OR 'surgical technician':ab,ti OR 'medical technician':ab,ti OR 'non-physician clinician':ab,ti OR 'allied health personnel':ab,ti OR 'hospital auxiliaries':ab, ti OR 'nurse clinician':ab,ti OR 'nurse practitioner':ab,ti OR 'advanced practice nurse':ab,ti OR 'clinical associate':ab,ti OR 'clinical nurse specialist':ab,ti OR 'non-physician clinician':ab,ti
**6**	'midwife'/exp OR 'nurse midwife'/exp OR 'nurse midwifery'/exp OR 'midwife':ab,ti OR 'midwives':ab,ti OR 'registered midwife':ab,ti OR 'community midwife':ab,ti OR 'certified nurse midwife':ab,ti
**7**	'nurse'/exp OR 'nursing staff'/exp OR nurse*:ab,ti
**8**	'nursing assistant'/exp OR 'auxiliary nurse':ab,ti OR 'nurses' aide':ab,ti OR 'nurse aid':ab,ti OR 'auxiliary midwife':ab,ti OR 'auxiliary nurse midwife':ab,ti
**9**	#1 OR #4OR #5 OR #6 OR #7 OR #8
**10**	'medical abortion'/exp OR ‘medical abortion’:ab,ti OR ‘medication abortion’:ab,ti
**11**	'incomplete abortion'/exp OR 'spontaneous abortion'/exp OR 'pregnancy termination'/exp OR 'termination of pregnancy':ab,ti OR 'miscarriage':ab,ti OR 'abortion':ab,ti OR 'postabortion care':ab,ti
**12**	'misoprostol'/exp OR 'mifepristone'/exp OR 'cytotec':ab,ti OR 'medabon':ab,ti OR 'RU486':ab,ti OR 'mifegyne':ab,ti OR ‘medical’:ab,ti OR ‘medication’:ab,ti
**13**	#11 AND #12
**14**	#10 OR # 13
**15**	#9 AND #14

CINAHL

**#**	**Search terms**
**1**	(MM "Physicians, Family") OR (TI (physician, primary care OR general practitioner OR family physician OR clinical officer OR non-specialist doctor)) OR (AB (physician, primary care OR general practitioner OR family physician OR clinical officer OR non-specialist doctor)
**2**	(MH "Alternative Health Personnel+")
**3**	(MH "Physician Assistants") OR (MH "Nurse Practitioners+")OR TI (physician assistant, clinical officer, nonphysician clinician, health officer, nurse practitioner, advanced practice nurse) OR AB (physician assistant, clinical officer, nonphysician clinician, health officer, nurse practitioner, advanced practice nurse)
**4**	(MH "Midwives+") OR (MM "Nurse Midwives") OR TI (midwife OR midwives) OR AB (midwife OR midwives)
**5**	(MH "Nurses+") OR TI nurse OR AB nurse
**6**	(MM "Nursing Assistants") OR (MM "Lay Midwives")
**7**	S1 OR S2 OR S3 OR S4 OR S5 OR S6
**8**	MH "Abortion, Spontaneous+") AND (MH "Abortion, Induced+") OR (MM "Abortion, Incomplete") OR TI (medical abortion OR medication abortion OR abortion OR postabortion

	care OR miscarriage) OR AB (medical abortion OR medication abortion OR abortion OR postabortion care OR miscarriage) AND (MM "Misoprostol") OR (MM "Mifepristone") OR TI (misoprostol or mifepristone) OR AB (misoprostol or mifepristone))
**9**	S7 AND S8

POPLINE (0 met criteria)

#	Search terms
	("abortion" or "miscarriage" or "postabortion care") AND ("misoprostol" OR "medical" OR “medication”)

Cochrane Database (0 met criteria)

#	Search terms
**1**	“abortion” OR “miscarriage”

Clinical Trials.gov

#	Search terms
**1**	“abortion”
